# Ethnobotanical studies on plant resources of Mangowal, District Gujrat, Punjab, Pakistan

**Published:** 2014

**Authors:** Muhammad Parvaiz

**Affiliations:** 1 *Department of Botany, Institute of Chemical and Biological Sciences (ICBS), University of Gujrat (UOG), Hafiz Hayat Campus (HHC), Gujrat 50700, Punjab, Pakistan*

**Keywords:** *Ethnomedicinalsurveys*, *Mangowal*, *Medicinaluses*, *Pakistan*, *Punjab*, *UOG*

## Abstract

**Objective: **This study was conducted to collect indigenous traditional knowledge about the medicinal plants and their uses in Mangowal, District Gujrat, Punjab, Pakistan.

**Materials and Methods: **The ethnomedicinal data were gathered through questionnaires and extensive personal dialogues with native individuals comprising 40 males and 20 females of diverse age groups between 50 to 80 years and also tabibs and hakims. Ethnobotanical investigations were directed from January 2013 to March 2013 under the acquiescence of university of Gujrat (UOG), Punjab, Pakistan.

**Results: ** About 40 plant species belonging to 22 families were investigated which are utilized by native people to cure various disorders and ailments such as asthma, ulcer, gonorrhea, piles, stomach pain, and skin diseases. Medicinal plants comprises of combination of active compounds which are side effect neutralizing and synergistic. Herbal medicines were prepared from different part of plants. Mostly used plant parts were leaves, fruits, barks, roots, seeds, and sometimes whole plant.

**Conclusion:** The results demonstrated that the area is rich in vegetation but remained botanically virgin and not explored extensively and intensively.

## Introduction

**Figure 1:**
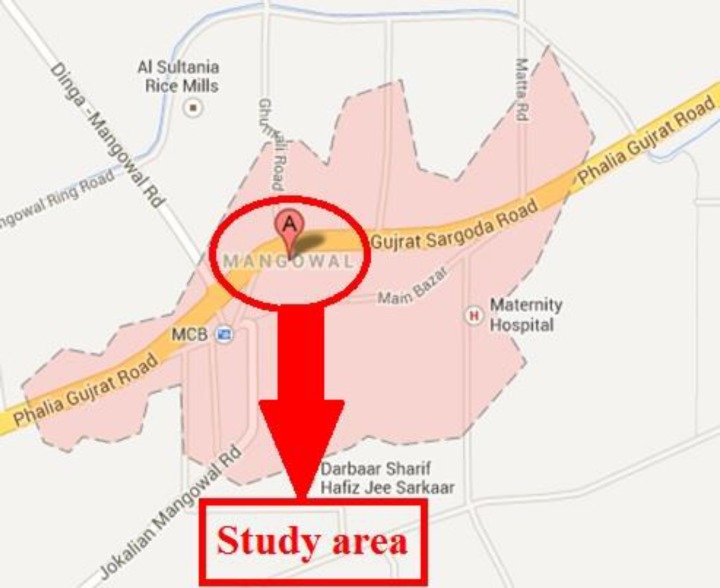
Map of the Mangowal, District Gujrat, Punjab, Pakistan

Mangowal is situated between the latitudes of 32°.495 North and longitudes of 73°.893 East and elevation is 254 m (836 ft) (Figure    1 ). Mangowal is 19 kilometer away from Gujrat towards west on Sargodha road. Mangowal is a business hub for surrounding villages due to its location as Gujrat Sargodha highway passes through the town. 

Gujrat is a primitive district of Pakistan positioned in the middle of two prominent rivers, the Jhelum River and the Chenab River. Because of its juxtaposition with the both rivers, the land is suitable for agronomy with rice (*Oryza sativa* L.) and sugar cane (*Saccharum officinarum* L.) as chief crops. It is constrained on the northeast by Jammu and Kashmir, on the northwest by the Jhelum River which divides it from Jhelum District, on the east and southeast by the Chenab River, separating it from the districts of Gujranwala and Sialkot, and on the west by Mandi Bahauddin District. District Gujrat covers an area of 3,192 square kilometers and contains three tehsils of Gujrat, Kharian, and Sarai Alamgir. The District Gujrat lies between 32° to 35° North latitudes and 73° 45' East longitudes. This district has moderate weather. During peak summer, the highest daytime temperature is up to 45°C, but the hot spells are relatively short due to the proximity to the Azad Kashmir Mountains. During the winter months the lowest temperature may fall below 2° C. The average rainfall on the Kashmir border is over 100 cm, at Kharian it is 75 cm, at Gujrat 67 cm, at Dinga 50 cm, and at Sarsal 48 cm (Hussain et al., 2010 ▶).

Ethnobotany is the logical study which plays an important role in understanding the vigorous relationships between human beings and plants (Rahman, 2013 ▶). Traditional aboriginal information is primitive as human civilization (Qureshi et al., 2007 ▶), but the term “ethnobotany” was first devised by the US botanist “John Harshberger” in 1895, to study the ethnomedicinal plants which are used by the earliest inhabitants of local areas (Mahmood et al., 2012 ▶). Nearly 80% of the inhabitants of emerging countries are still dependent on traditional aboriginal remedies for their primary healthcare (Shah et al., 2013 ▶). Pakistan has a high diversity of plants that are being used by local communities for medicinal purposes. Suitable usages of these plants are commonly practiced at the community and end user level. Many studies have been conducted on the ethnobotany of medicinal and other useful plants throughout the world (Khan et al., 2013 ▶). 

Ethnobotanical studies in different areas of Pakistan have also been carried out (Qureshi et al., 2011 ▶). In many developing countries, ethnomedicinal plants have not been well studied and exploited, tested, or documented. Most of the information is still in the hands of traditional hakims and knowledge of hakims is either lost or passed to next generation orally (Amiri et al., 2013 ▶). Pakistan has about six thousand species of wild plants of which six hundred are medicinally used by both animal and human to treat ailments through the use of traditional medicinal plants. In most instances, certain plant species are considered specific for a particular illness, but occasionally, plants have various usages (Khan et al., 2012 ▶). 

**Figure 2:**
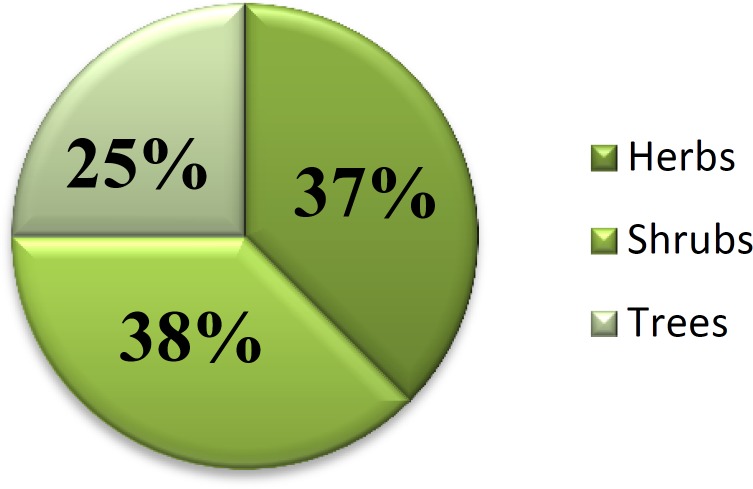
Percentage of age of plants according to habit

**Figure 3:**
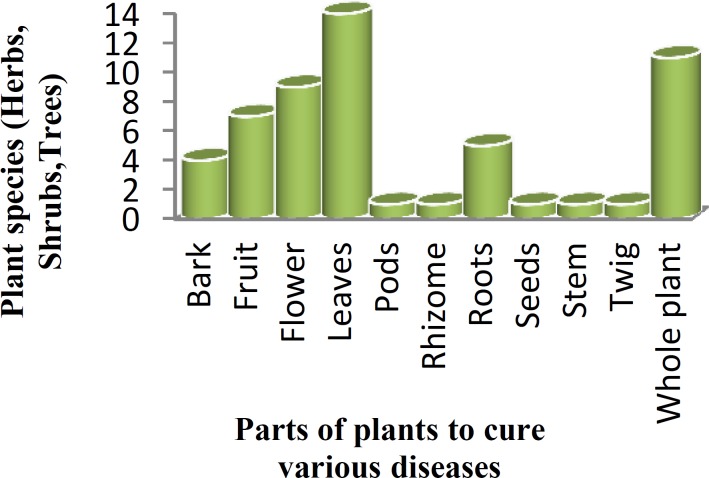
Parts of plant species to treat different diseases

## Materials and Methods

**Surveys**

Many surveys were conducted to collect the basic information regarding the Mangowal, District, Gujrat, Punjab, Pakistan. The ethnomedicinal surveys were conducted from January 2013 to March 2013 with the permission of University of Gujrat (UOG), Pakistan.

**Interviews with local inhabitants**

The ethnomedicinal data were gathered through questionnaires and extensive personal interviews of local inhabitants including 40 men and 20 women of different age groups between 50 to 80 years (Table 5). Mostly old people were interviewed as they have a lot of information about the medicinal uses of different local plant species. Tabibs, Hakims and Pansars were also consulted as they have more awareness about medicinal properties and proper usage of plants. Open ended and partially structured inventories were filled during the interviews of local inhabitants of study area. Data were collected on different aspects by asking questions such as: Do you have information about the medicinal plants of your area? If your answer is yes, then name them. What are the uses of these important medicinal plants? How these are utilized and for which diseases? Which parts of plant species are used for medicinal purposes? The interviewer were asked questions in Punjabi (Mother language of Punjab, Pakistan) and Urdu (National language of Pakistan) because ease of local inhabitants which are uneducated and English language is not understandable in most cases. 

**Samples collection, identification and preservation**

Plant samples were collected from study area and arranged alphabetically with their exact nomenclature, family name, botanical name, common name, habit, used parts, and medicinal uses. Plant specimens were pressed, dried, and mounted on the herbarium sheets. All collected samples were identified with the help of available literature of (Nasir et al., 1971-1995). After identification, specimens were submitted to the Herbarium of the department of Botany, University of Gujrat (UOG), Punjab, Pakistan for future references.

## Results

The present research study is based on the traditional knowledge of most commonly used medicinal plants of Mangowal, District Gujrat, Punjab, Pakistan. A total of 40 plant species belonging to 22 families are reported from study area (Tables 1, 2, 3, and 4). These commonly used plant species are arranged in alphabetical order followed by their family name, botanical name, common name, habit, used parts, and their uses. Research study was focused on the traditional medicinal uses of that area. During these work, 40 males and 20 females of different age groups between 50 to 80 years and also 5 tabibs and 10 hakims were interviewed. Ethnomedicinal uses and data about treatment of different disorders and ailments based on the information were collected from local inhabitants using questionnaires.

**Table 1:** List of therapeutic herbs of Mangowal, District Gujrat, Punjab, Pakistan

**Sr#**	**Botanical name**	**Common name**	**Family**	**Habit**	**Part used**	**Medicinal uses**
**1**	*Abutilon indicum* (L.) Sweet	Peeli booti	Malvaceae	Herb	Flowers and leaves	Used for treatment of bleeding piles, diarrhea, toothache, and inflammations.
**2**	*Asparagus racemosus* Willd.	Satmuli	Liliaceae	Herb	Roots	Antispasmodic, carminative, diuretic, dyspepsia, diarrhea, and dysentery.
**3**	*Calendula officinalis* L.	Gutta	Asteraceae	Herb	Flowers	Used to treat healing wounds, injuries. The plant is valued for treatment of burns and ulcers.
**4**	*Carthamus oxyacantha* M.Bieb.	Poli	Asteraceae	Herb	Whole plant	It is used to treat jaundice, male infertility, and also cure scabies.
**5**	*Cichorium intybus* L.	Kasini	Asteraceae	Herb	Whole plant	Used for the purification of blood and liver.
**6**	*Conyza bonariensis* (L.) Cronquist.	Dhania booti	Asteraceae	Herb	Whole plant	Plant has diuretic properties and is effective against diarrhea and dysentery.
**7**	*Cynodon dactylon* (L.) Pers.	Bham grass	Poaceae	Herb	Whole plant	Plant is used to treat wounds, kidney problems, and also bronchial disorders.
**8**	*Cyperus rotundus* L.	Deelie	Cyperaceae	Herb	Leaves, rhizomes	Decoction of leaves is used as antihelmintic and for skin disorders.
**9**	*Foeniculum vulgare* Mill.	Sounf	Apiaceae	Herb	Fruit	It is used for vomiting, dyspepsia, and heart burns.
**10**	*Helianthus annuus* L.	Suraj muki	Asteraceae	Herb	Flowers and seeds	The flower is pungent, anthelmintic, and also used for treatment of asthma, ulcer, and skin diseases.
**11**	*Mentha longifolia* (L.) L.	Jangli podina	Lamiaceae	Herb	Whole plant	Plant is used to cure fever and gas problem in stomach.
**12**	*Parthenium hysterophorus* L.	Gandi booti	Asteraceae	Herb	Whole plant Leaves	It is used for to remove toothache and kill tape worms.
**13**	*Saccharum spontaneum* L.	Sarrout	Poaceae	Herb	Whole plant	Used for treatment of abdominal pain and improvement of appetite.
**14**	*Sonchus asper *(L.) Hill.	Mai buddi, dodak	Asteraceae	Herb	Whole plant	Diuretic, sedative, and also useful for cough, asthma, and burns.
**15**	*Tagetes patula* L.	Genda	Asteraceae	Herb	Flowers and leaves	Antifungal. Leaves are useful to cure piles, fever, earache, and muscular pain.

**Table 2:** List of therapeutic shrubs of Mangowal, District Gujrat, Punjab, Pakistan

**Sr#**	**Botanical name**	**Common name**	**Family**	**Habit**	**Part used**	**Medicinal uses**
**16**	*Achras zapota* L.	Chiku	Sapotaceae	Shrub	Bark, fruit & seeds	Diuretic and useful in fever.
**17**	*Agave americana* L.	Kantala	Agavaceae	Shrub	Leaves	Used in dropsy and toothache.
**18**	*Calotropis procera* (Aiton) W.T.Aiton	Aak	Asclepiadaceae	Shrub	Leaves	It is used to cure asthma and snake bite.
**19**	*Carissa carandas* L.	Karoonda	Apocynaceae	Shrub	Whole plant	Stomach disorder and skin infection.
**20**	*Euphorbia milii* Des Moul.	Common euphorbia	Euphorbiaceae	Shrub	Flowers	Used for treatment of cancer.
**21**	*Hibiscus rosa-sinensis* L.	Gurhal	Malvaceae	Shrub	Flowers	Paste is used to reduce burning sensation.
**22**	*Mimosa pudica* L.	Chui mui	Leguminosae	Shrub	Roots and leaves	Asthma, inflammation, and fatigue.
**23**	*Murraya exotica* L.	Marwa	Rutaceae	Shrub	Leaves and roots	Used for skin diseases, dyspepsia, diarrhea, and dysentery.
**24**	*Murraya koenigii* (L.) Spreng.	Karry pata	Leguminosae	Shrub	Leaves	Asthma, blood diseases, dysentery, and leprosy.
**25**	*Nerium indicum* Mill.	Oleander	apocynaceae	Shrub	Flowers and roots	Used for treatment of abortion and cancer.
**26**	*Ocimum basilicum* L.	Niazboo	Lamiaceae	Shrub	Leaves	It is used for skin infection, cold, and cough.
**27**	*Rosa indica* L.	Gulaab	Rosaceae	Shrub	Flowers	Used for asthma and eye infection.
**28**	*Solanum nigrum* L.	Kainch mainch	Solanaceae	Shrub	Fruit and leaves	Used for gastric troubles, ulcer, and jaundice.
**29**	*Withania coagulans* (Stocks) Dunal	Choota Aak	Solanaceae	Shrub	Fruit	Plan is diuretic, sedative, and also useful for cough, asthma, and burns.
**30**	*Withania somnifera* (L.) Dunal	Aak san	Solanaceae	Shrub	Whole plant	Used for asthma, constipation, eye infections, rheumatic disorders, and ulcers.

**Table 3:** List of therapeutic trees of Mangowal, District Gujrat, Punjab, Pakistan

**Sr #**	**Botanical name**	**Common name **	**Family**	**Habit**	**Part used **	**Medicinal uses**
**31**	*Acacia nilotica* (L.) Delile	Keekar	Leguminosae	Tree	Bark and pods	Used for treatment of gonorrhea.
**32**	*Azadirachta indica* A.juss.	Neem	Meliaceae	Tree	Leaves	Used for blood purification, cough, and diabetes.
**33**	*Bauhinia purpurea* L.	Kachnar	Fabaceae	Tree	Whole plant	Plant is antibacterial, antidiabetic, and anti-inflammatory.
**34**	*Bombax ceiba *L.	Sumbal	Malvaceae	Tree	Flowers	Blood purification, gonorrhea, and snake bite.
**35**	*Carica papaya* L.	Papeeta	Caricaceae	Tree	Fruit	It is used for digestive problems and abortion.
**36**	*Dalbergia sissoo *DC.	Tali	Mimosaceae	Tree	Bark	Used to cure nose bleeding.
**37**	*Ficus benghalensis* L.	Boher	Moraceae	Tree	Bark and fruit	Used for treatment of gonorrhea.
**38**	*Melia azedarach* L.	Dhreak	Malvaceae	Tree	Fruit and leaves	Skin diseases and skin infections.
**39**	*Polyalthia longifolia *(Sonn.) Thwaites	Ultha ashok	Annonaceae	Tree	Leaves, roots and stem	It is used to cure diabetes, skin diseases, and fever.
**40**	*Pongamia glabra* Vent.	Sukh chain	Fabaceae	Tree	Twigs	Twigs used for diseases and infections of teeth.

**Table 4:** Plant species of Mangowal, District Gujrat, Punjab, Pakistan arranged according to their families

**Sr No.**	**Name of Families**	**Plant species of (Table 1. 2. 3)**
**1**	Agavaceae	1
**2**	Annonaceae	1
**3**	Apiaceae	1
**4**	Asclepiadaceae	1
**5**	Apocynaceae	2
**6**	Asteraceae	8
**7**	Caricaceae	1
**8**	Cyperaceae	1
**9**	Euphorbiaceae	1
**9**	Fabaceae	2
**10**	Lamiaceae	2
**11**	Leguminosae	3
**12**	Liliaceae	1
**13**	Malvaceae	4
**14**	Meliaceae	1
**15**	Mimosaceae	1
**16**	Moraceae	1
**17**	Poaceae	2
**18**	Rosaceae	1
**19**	Rutaceae	1
**20**	Sapotaceae	1
**21**	Solanaceae	3

**Table 5:** Meetings with native people of Mangowal, District Gujrat, Punjab, Pakistan

Age groups of Males/females	Quantity of interviewees
**50-60 (10 males)**	10
**63-69 (20 males/5 females)**	25
**70-75 (10 male hakims/7 females)**	17
**75-80 (8 female)**	8
Total	60

## Discussion

The present research aimed to provide medicinally important indigenous information about traditional plants. The main focus of this study was to collect information about the medicinal uses of plants, by way of their uses, and to some extent the traditional medicinal plants used by local inhabitants that are used as remedies to treat themselves. The basic health care facilities are absent therefore the people of the area principally depending on medicinal plants for the treatment of various diseases and disorders. It was observed that old people are more liable to the use of ethnomedicinal plants as primary health care in contrast with new peers. The plants were categorized according to their habit into three forms, namely herbs, shrubs, and trees. The proportion of utility of remedial plants in study area was 37% herb species, 38% shrub species, and 25 % tree species (Figure 2). 

About eighty percent of the inhabitants of local areas depend on the medicinal plants for the manufacturing of drugs that are used for the treatment of different diseases (Joudi et al., 2010). For example, *Acacia nilotica* (L.) Delile and *Carthamus oxyacantha* M. Bieb. are used for the treatment of hepatitis and jaundice (Mahmood et al., 2011 ▶). *Calotropis procera* (Aiton) W.T. Aiton was used for the treatment of asthma and snake and insect bite (Abbasi et al., 2005). *Solanum nigrum* L. locally called as ‘Kainch mainch’ is used for the treatment of jaundice (Mahmood et al., 2011 ▶). *Tagetes patula* L. plant has antifungal activity and is used to cure fungal infections in human beings (Rahman. 2013 ▶). Similarly, different parts of plant species (herbs, shrubs, and trees) such as bark, rhizome, roots, stem, fruits, flowers, leaves, and pods are used to cure various diseases, disorders and ailments (Figure 3).
